# Stress Whitening as an Observation Method of Residual Stress in MABS Polymer Material through the Example of Holding Pressure in an Injection Molding Process

**DOI:** 10.3390/polym12122871

**Published:** 2020-11-30

**Authors:** Paweł Brzęk, Tomasz Sterzyński

**Affiliations:** Division of Polymers, Institute of Materials Technology, Poznan University of Technology, 61-138 Poznań, Poland; Tomasz.Sterzynski@put.poznan.pl

**Keywords:** residual stress, residual stress measurement, stress whitening, MABS, photoelasticity, holding pressure

## Abstract

The effects such as warpage, dimensional instability and environmental stress corrosion, due to the presence of residual stresses in polymeric products, are strongly dependent on injection molding conditions. The holding time and holding pressure belongs to most important processing parameters, determining the dimensional stability and properties of injected goods. A new procedure based on a visualization technique was applied, where the levels of residual stresses of the samples were estimated. The experiments were performed for samples produced of translucent methacrylate acrylonitrile butadiene styrene (MABS), a commodity polymer with a high transparency, necessary for the optical visualization of the stress whitening. The samples produced by injecting molding were deformed to a constant elongation, to observe the dependent stress whitening effect subsequently used to evaluate the stress distribution. It was found that depending on the value of the injection holding pressure, various levels of residual stress and its distribution may be observed in MABS samples. These measurements conformed that the applied optical method is an easy-to-perform technique. The possibility to detect the residual stresses over the whole cross-section of the transparent product, without the necessity for local stress determination, is another significant advantage of this investigation procedure.

## 1. Introduction

Injection molding is among the most used processing techniques for the production of goods in all industrial branches, such as house holding, house building, transportation, every-day life, electronic and electro-techniques. The creation of goods which are mostly ready to use, even those very complex in shape, in a relatively short processing time, is a well-known capability of this processing technique. Several requirements have to be met though, if products with the desired quality are to be fabricated.

**Figure 1:**
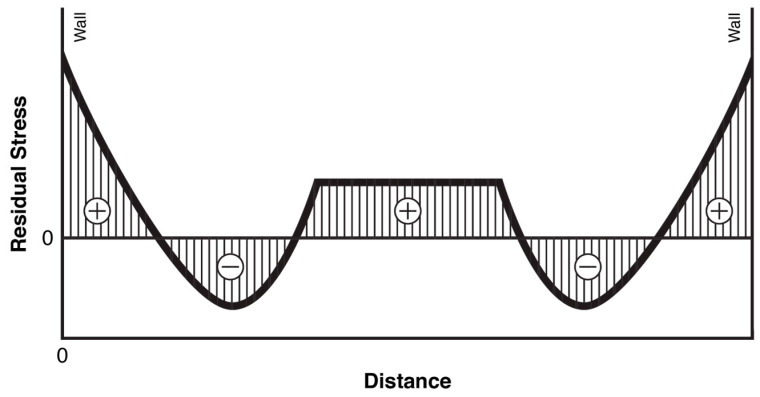
Scheme of residual stress in a cross-section of an injection molded part [2].

Residual stresses in injection molded products may significantly influence their dimensional tolerances, self-deformations and stress corrosion [1]. During processing the crucial impacts on stress formation in the products are molten polymer flow by cavity filling and thermal and pressure history. Two basic mechanisms of stress formation may be considered, i.e., flow induced stresses and thermally induced stresses, a topic comprehensively described in the literature [2]. Both of those mechanisms result in the following stress distribution: a very thin tensile layer on the surface; a compressive stress layer underneath the surface; and tensile stresses in the center (Figure 1).

The processing parameters usually rarely contribute to the overall shape of the residual stress distribution over the cross-section of the product, but they may have large impacts on the values of stress, and thicknesses for specific layers and the gradients between them. The most important parameters influencing the stress profile are holding time and pressure, injection speed and mold temperature [3,4,5,6,7]. A higher mold temperature leads to lower stress values—both compressive and tensile—due to higher stress relaxation in these thermal conditions. By increasing the injection speed, usually the reduction of stress may be observed; however, a very slow filling rate may lead to a reversal of the stresses, resulting in a slight stretch in the center and a compression closer to the wall of the mold cavity. An increase of holding pressure and longer holding time affect the values of stresses, and slightly change the tensile to compressive stress at the center of the molding [8]. In addition, the geometry of the molded parts also have great impacts on stresses, particularly the wall thickness and the length of the flow path [9].

Over the years, several methods of residual stresses measurement by polymeric materials have been developed. Some of them were initially used for metal testing purposes and some are exclusive for the study of polymer materials. Most popular in scientific polymer studies is the layer removal method; however, in industry applications of photoelasticity and chemical probes are more common. Other methods include hole-drilling, indentation and X-ray measurements.

The layer removal method consists of milling a thin layer of material and measurement of deformation caused by imbalance in the stress equilibrium of the product. By knowing the mechanical properties of the material, it is possible to calculate the stresses that were in the milled layer. By repeating this procedure, it is possible to determine the stress distribution on the cross-section, which is a great advantage compared to other methods. This method is complex from the mathematical point of view, and the experiments are very susceptible to their conditions [10,11]. In addition, the method is limited by the shape of straight beams of uniform thickness. Hole drilling and measurements of local deformation by means of strain gauges are often used for the determination of residual stresses in various polymeric products [12].

The photoelastic method is based on the phenomenon of birefringence of polymeric materials. By analyzing the polarized light passing through the sample it is possible to infer the stress distribution and its level. This method has been mainly developed for stress analysis of model construction and stress imaging in metal elements [13]. As a non-destructive and very quick method to perform, it is often used in the quality control of optical elements [14]. The measurement of the average stresses on the thickness of the sample may be performed by means of this method. The results are, however, influenced by the macromolecular orientation, which may cause interpretation problems [2]. This method is limited to transparent materials only, which makes it very hard to use in cases of crystalline polymers or composites.

The chemical probe method is based on the determination of environment stress cracking. For specific polymeric materials and liquid chemical compounds, a relation is established between stress and time to craze or crack. In order to acquire values of stresses, experimental data are needed as a reference. If those requirements are met, then the method is very simple and quick to use as a quality control. The disadvantage of this technique is that it provides information only about tensile stress on the surface of a specimen.

Throughout the diversity of residual stress testing methods, there is no universal method that could be applied to any case. For example, the X-ray method applies only to crystalline materials, whereas photoelasticy limits itself to transparent materials. The layer removal method gives average information about single removed layers. The photoelastic method sums the stress in the direction of light’s passage, whereas the chemical probe limits itself only to the surface. There is no comprehensive method for residual stress measurements. It is therefore important to develop new test methods, giving a wider range of possibilities.

The method proposed in this article is based on the stress whitening of polymeric materials. In earlier studies [15], stress whitening as a mechanical measurement method was considered as low accuracy, but with some merit. It is extremely simple and could be used in three-dimensional problems [16], in contrast to the photoelastic method operating in high tensile stress. It was used only as a method of calculation for local stresses distribution caused by an external force [17], but never for establishing the residual stress.

Various macro-scale optical techniques were used to study the intensity of stress whitening. A simple and reliable method involves a single light source (laser or a lamp) and a single detector. This approach was successfully carried out in real-time measurement of whitening during the deformation [16,18]. Better results could by acquired using a CCD (Charge-Coupled Device) camera to obtain photos and subsequently analyzing them [19,20].

Stress whitening is a phenomenon visible on the macro-scale, as a color change under a certain load. At the micro-scale, it is an effect of microcracks or reorientation of polymer chains. In ABS and MABS materials, the microcrack formation mechanism predominates. When the molecular weight is lower than a certain critical value, the polymer chains do not form a dense, entangled network, and under a static load, they have a tendency to break in a brittle way. Above this critical value, during deformation, the chain network is stretched and micro-voids form between the chains. Due to the entanglement of the network, the material still retains its integrity. With further increases of deformation, the voids transform from closed-cell structures to open-cell structures, which result in clear cracks on the material [21,22]. Accumulations of microcracks and scratches cause the dispersion of visible light, which appears as whitening of the material. Cracks come prior to forming surface defects and structural errors; their size and distribution in the structure depends on the speed of deformation [23].

In this paper, a new approach to residual stress measurements will be presented. As the method is based on visible light detection and stress whitening phenomena, a transparent MABS material was chosen for this purpose. As a variable for producing test specimens, holding pressure in injection molding was measured. Its role in residual stress formation is well known, and when changed over a wide rage can give considerable differences in the stress of moldings.

## 2. Materials and Methods

### 2.1. Sample Preparation

**Figure 2:**
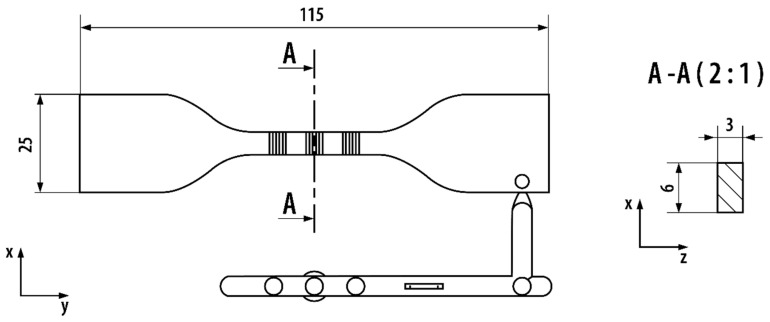
Scheme of the mold cavity and runner (length in millimeters).

The MABS Toyolac 900-352 (Toray Industries Inc., Tokyo, Japan), a transparent polymer similar to ABS, was used in the experiments. Tensile samples (Figure 2) were produced by injection molded by means of an Engel E-mac 50 (Engle Austria GmbH, Schwertberg, Austria) fully electric machine. The electric drive of the machine ensures substantial repeatability of the process conditions in comparison to conventional hydraulic injection molding machines. Injection process parameters are presented in Table 1.

The degree of mold cavity filling during the injection phase was set at about: 98–100% (in the absence of holding pressure, 1 in 10 samples was short-shot). The holding time was estimated based on the mass of the moldings. The holding pressure (*P_h_*) was chosen as follows: low (20 MPa), at which the residual stresses should be at minimum; high (100 MPa), highest possible for this mold, just below the value at which the molded part has difficulties with ejection; middle (60 MPa), at the center between low and high.

### 2.2. Photoelastic Observation

For validation of the stress whitening method, a photoelastic technique was used. The observations of the samples were carried out in polarized light on OPTA-TECH SK series microscope (Opta Tech Sp. z o.o., Warsaw, Poland) with the camera Meiji Techno HD2600T (Meiji Techno CO., LTD., Saitama, Japan). The angle between the polarizer and analyzer was set to 90°, and the sample was at 45° to them. This arrangement unveils principal stress in the best contrast. The investigation’s focus was only on the central part of the sample due to the constant one-way flow of the material during the injection phase and the ability to directly compare the images with the results obtained by stress whitening method. As the color of fringes was constant through the lengths of the samples, the 2D plane was averaged out to a 1D line for every image.

For image analysis a three-fringe photoelasticity (TFP) technique was used, which is well described in the literature [13,24]. Each pixel from an image was compared, in three-dimensional RGB space, to the calibration table in order to find a closest match (minimum *e*):

(1) $$e=\sqrt{{\left(R_{e}-R_{c}\right)}^{2}+{\left(G_{e}-G_{c}\right)}^{2}+{\left(B_{e}-B_{c}\right)}^{2}},$$

where the *R*, *G* and *B* stand for red, green and blue components of the pixel; subscript “*e*” refers to experimental data and “*c*” to the calibration table. The calibration table was determined from photos of the stressed samples and adjusted using theoretical curves described by Sørensen [25].

### 2.3. Tensile Test and Stress Whitening Observations

For further calculations a stress–strain curve was mandatory. For this purpose, a tensile test was carried out on an Instron 4481 universal testing machine (ITW Instron, Norwood, MA, USA), with a traverse rate of 20 mm/s. For every holding pressure, 5 samples were tested up to final break. The average values of mechanical properties for all series were used as references for calculations as stress-strain curves *R*(*ε*).

The tensile stress machine was used also for stretching of the samples for the main experiment of stress whitening measurements. The goal was to obtain a series of samples showing the evolution of stress whitening during the stretching. For that purpose, the testing machine was set to stop at a certain elongation *ε_n_*. The stress whitening started to appear in the samples at an elongation of about *ε* = 3.0%, and the maximum of its intensity was reached by *ɛ* = 5.0%. This was the range allowing one to analyze the whitening during stretching of the material. An elongation step of *Δε* = 0.2% was selected as multiple elongation values. For every holding pressure (*P_h_*) and elongation (*ε_n_*) combination, 3 samples were produced.

Optical observations of the samples were carried out by means of transmitted light using an OPTA-TECH SK series microscope (Opta Tech Sp. z o.o., Warsaw, Poland) equipped with a Meiji Techno HD2600T camera (Meiji Techno CO., LTD., Saitama, Japan). Dark room conditions were applied to ensure minimum redundant light, and the only source of light was under the sample when the lens of the microscope was exactly above. The light passing through the sample was scattered upon stress whitening; thus, the whitening phenomenon is visible on the photograph as darkness. The auto-adjustment of camera settings was disabled. With the use of a light-histogram analyzer built into the camera’s software, none of the samples were underexposed nor overexposed; one setting was used for all samples’ images.

For the quantification of stress whitening the microscope’s images were processed using ImageJ (ver. 1.52a) software to obtain the grey scale values (*G*). The grey scale was averaged on the length of the samples (in the y axis) and presented as a curve of *G*(*x*), where *x* is a distance from the edge (max *x* = 6 mm) and values of *G* are in range from 0 to 255.

### 2.4. Calculation of Residual Stress

**Figure 3:**
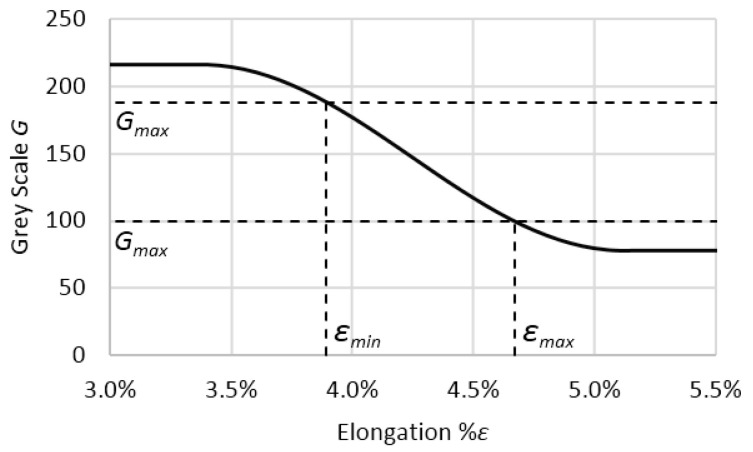
Grey scale to elongation relationship. Dashed lines represent limits of the function used in further calculations.

The assumptions were made that a uniform stress state is present in the cross-section of the sample during uniaxial stretching and that the intensity of stress whitening is related to elongation. As the grey scale acquired from microscope images can describe stress whitening intensity, a correlation between grey scale and uniaxial elongation is the basis for evaluating the residual stresses in the samples. This function was prepared from the middle section of samples injection molded with minimum holding pressure (*P_h_* = 20 MPa); it was assumed that the lowest stress values would be preserved [3,7]. On this basis, an empirical function was proposed to describe the relation:

(2) G=A sin(Cε+D)+B,

where *A*, *B*, *C* and *D* are the values chosen to best fit the real curve. On account of the residual stress present in the reference sample, those values still needed calibration. This was done through finding the minimum of the sum of the standard deviations of the residual stress results. The final values are given in Table 2. This function describes the part of the curve where the grey scale changes as a function of elongation. Below and above some values of elongation, grey scale is constant and has no relevant information for this analysis, so the function was limited by *G_max_* and *G_min_*, corresponding to elongation values *ε_max_*, *ε_min_* (Figure 3). It has to be kept in mind that all of those values describing the function of grey scale to elongation (*G-ε*) curve may be applied only to images created in specific light conditions, and in every case the curve should be properly calibrated to new image settings and sample behavior.

A limited range of the *G-ε* curve was used to transform the grey scale *G*, obtained from images, into a graph showing the grey scale elongation (*ε_G_*) of any point on the *x* axis. The graph is limited by the *ε_max_*, *ε_min_* values, and by the comparison of a certain number of images, taken with different *ε_n_*, it is possible to obtain information for the whole *x* axis. On this basis it is possible to estimate the residual stress *R_G_* at any point correlated with this elongation:

(3) $$R_{G}=R\left(\varepsilon_{G}\right)-R\left(\varepsilon_{n}\right),$$

where *R*(*ε*) is the value of stress obtained from the stress–strain curve at *ɛ* elongation.

## 3. Results

**Figure 4:**
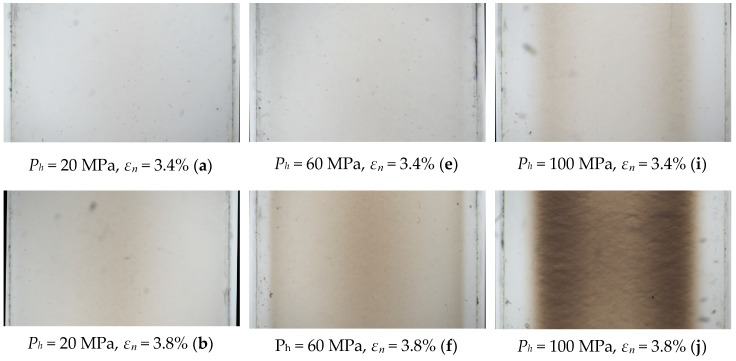

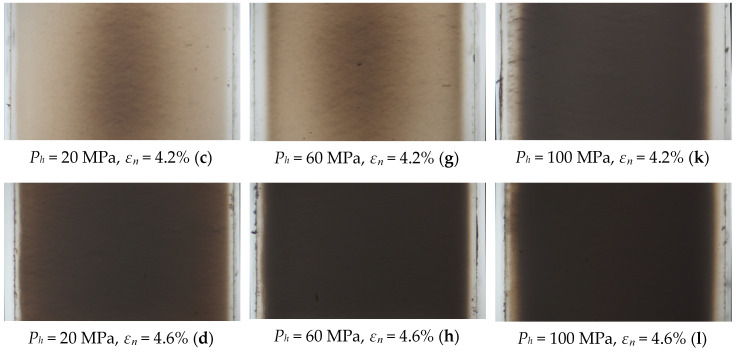
Examples of microscopic photographs displaying differences of stress whitening in relation to holding pressure (*P_h_*) and elongation (*ε_n_*) (width of samples: *x* = 6mm).

The microscopic photographs of samples produced by various injection molding holding pressures (*P_h_*) and uniaxially deformed to defined elongation *ε_n_*_,_ are presented in Figure 4. Due to the arrangement of the optical system with light transmitted by registration, the stress whitening is visible as darkness on the images.

**Figure 5:**
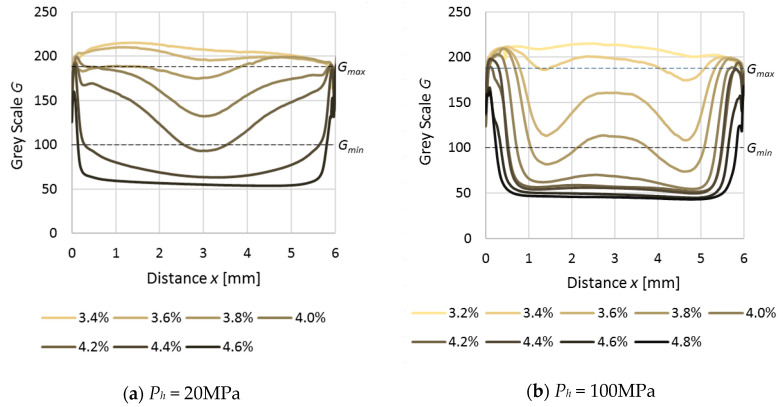
Grey scale *G* in relation to distance from the edge for samples produced with different holding pressures: (**a**) 20 MPa and (**b**) 100 MPa.

The graphical presentation of the average value of the grey scale (*G*) on the width of the samples may be seen on Figure 5. Clearly visible are the differences of the whitening effect on the samples produced by various packing pressures (*P_h_*). For samples formed with a low holding pressure *P_h_* = 20 MPa, the whitening due to the uniaxial deformation is relatively uniform, at least in the cross-sections—only a darker band at the center and thin bright layers on outer parts may be seen. On the contrary, for samples produced with a higher holding pressure (*P_h_* = 100 MPa) the appearance of the dark zone in the central part is evident, followed by its extension towards the edges of the samples for higher elongations of sample; there was well visible alteration in whitening at the centers of the samples. Moreover, the differences of elongation value, required for the appearance of whitening, are observable; i.e., for *P_h_* = 20 MPa, the elongation range is between 3.8% and 4.6%, and for *P_h_* = 100 MPa, between 3.2% and 4.8%. For the pressure *P_h_* = 60 MPa an intermediate effect was observed. The obtained results clearly show subtle irregularities in the grey scale (*G*) of photographs. The obtained *G* values should be symmetrical toward the center of the sample, but all of them show slightly higher *x* values; a probable explanation for this effect is non-uniform microscope light.

**Figure 6:**
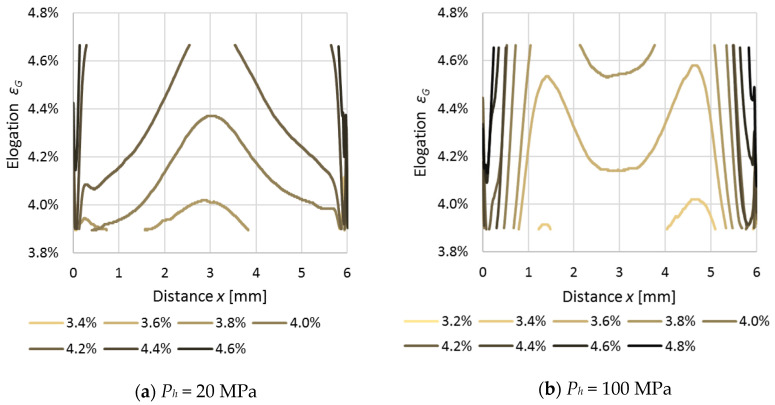
Elongation evaluated from grey scale (*ɛ_G_*). Samples produced with different holding pressures: (**a**) 20 MPa and (**b**) 100 MPa.

Using the calibration curve—grey scale to elongation (*G-ε*) (2)—the gray scale *G* profiles were transformed into elongation *ε_G_* curves; the results are presented in Figure 6. in a form of elongation *ε_G_*, evaluated on the basis of whitening, which does not present the same values as *ε_n_*. The authors have made an assumption that the differences were due to an existence of former residual stresses in the samples leading to slight local deformations. By overlapping those deformations via uniaxial elongation *ε_n_* of the samples, a total deformation *ε_G_* was achieved.

**Figure 7:**
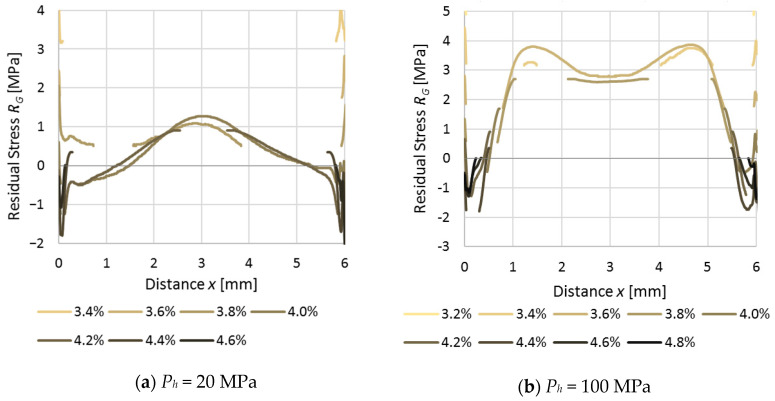
Residual stress calculated from grey scale (*R_G_*). Samples produced with different holding pressures: (**a**) 20 MPa and (**b**) 100 MPa.

**Figure 8:**
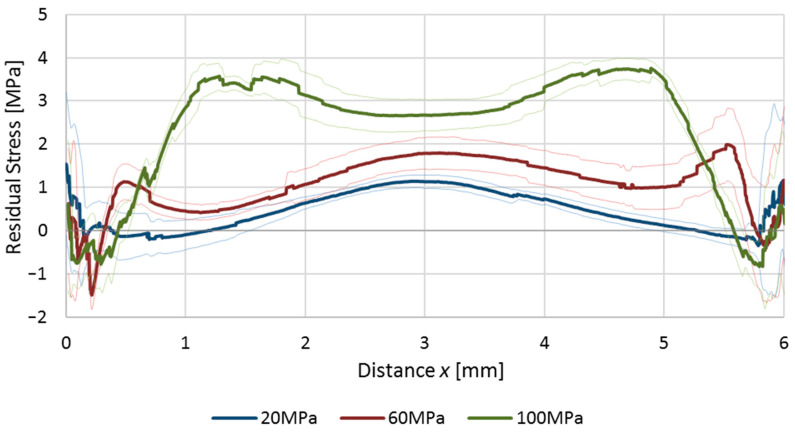
Residual stress curves obtained via stress whitening method for samples produced with different holding pressures (*P_h_*). Thin lines represent standard deviation.

By using Equation (3), the value of *ε_G_* may be evaluated as to the residual stresses presented in Figure 7. Due to the experimental nature of such study, curves obtained from different *ε_n_* values do not always match together ideally; nevertheless, the overall shape of the residual stress could be deducted. In both of the series (*P_h_* = 20 MPa and *P_h_* = 100), there were multiple, fragmented lines on the edges of the samples. They are the effect of a very narrow layer near the wall that did not get whitened during stretching. The final profiles of residual stress (Figure 8.) were achieved as a combination of experimental curves; thus, the shape of the curve may have a not-smoothed form.

The resulting residual stress profiles are in agreement with the literature values [2,3,26]. It was found that samples produced by a holding pressure (*P_h_* = 100 MPa) had high levels of tensile stresses at their centers. At a distance of 1 mm measured from the edges of the samples, the tensile stress was decreasing and finally transformed into a compressive state, with a slight increase in the values close to the external wall. For samples produced with *P_h_* = 60 MPa, a lower tensile stress at the central part may be seen, where the near-wall compression zone is thinner compared with samples made with *P_h_* = 100 MPa. The same tendency was observed for samples produced with *P_h_* = 20 MPa; the overall stress curve was shifted to lower values and the difference between tensile and compression near the wall of a sample disappeared. The stress measured directly near the wall has exceptionally high standard deviation, so the measured values are not fully trustable.

**Figure 9:**
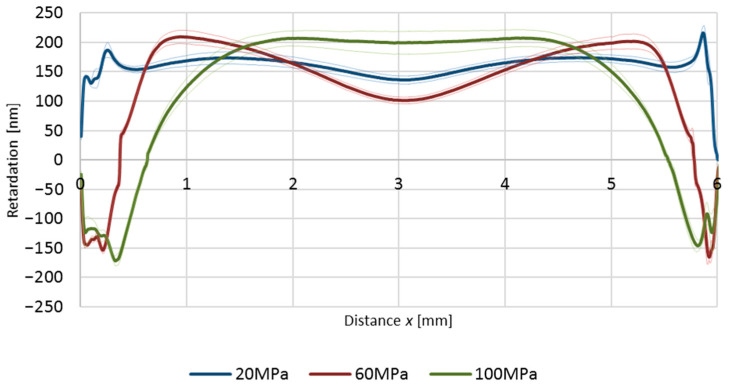
Calculated retardation of light passing through samples prepared at different holding pressures (*P_h_*). Thin lines represent standard deviation.

Certain characteristics of whitening residual stress curves are visible for those obtained by the elastooptic method (Figure 9.), but the direct translation between both is not possible. With the increasing of holding pressure, the width of the near-wall compression zone rose; however, differences between values of retardation of the central zone are not evident.

This may be due to the principal difference between the methods in the interpretation of the stress state through the thickness of the material. It was proved that in the tested samples, in the *x* direction, compressive stress occurs in the near-wall zone and tensile stress is in the central zone. The same applies to the perpendicular direction in which the samples were optically registered. In the elastooptic method, if the light beam passes through material which is in tensile state, the retardation has positive values; if the state is compressed, negative. However, if the light passes through layers of material which have different stress states (as in our case: compression–tensile–compression) the result is a sum of the retardation of all layers. On the other hand, the tensile stress in the sample is visible as first and may cover the compressive stresses occurring in the cross-section. This effect may explain small differences of retardation in the central parts of samples prepared by different holding pressures (*P_h_*).

## 4. Discussion

There is a visible relationship between our results with those from the elastooptic method, following the theoretical distribution of the residual stresses in injection molded parts. It was observed that the level of holding pressure has a significant impact on the tensile residual stresses at the central part of a sample, and on the thickness of the compressed near-wall layer.

This was a first attempt of using the white stress method for calculating the residual stresses, and it still requires an improvement. The mathematical approach to the method is very simple and ignores many nuances, such as lowering the strength of already whitened parts of the cross-section, when calculating the stresses in the remaining parts. A greater challenge is the acquiring of a reliable gray scale to elongation (*G-ε*) calibration curve. Its shape depends on many factors, such as: sample thickness; material transparency; microscope illumination conditions; CCD sensor and its settings. Another technical problem is the repeatability of both the photography conditions and sample preparation process. For one measurement of the residual stress state, a minimum of 15 identical samples are required. A shortcoming of the method is also its only being suitable for transparent materials showing stress whitening, so this may restrict its applicability, especially for not sufficiently transparent materials.

Apart from the disadvantages, the method has also many advantages not seen in other residual stress measurement techniques. It is not limited to 2D analysis; with properly prepared samples, the observation of parts from three different directions is fully achievable, with analysis of stress distribution in the entire sample volume. The method is not limited to simple forms of the products, such as beams, plates and discs; evidently, any shape of the product may be investigated. Without complex calculations the weakest points of the molded part structure, and the influences of residual stresses on their strength, can be visualized and analyzed. Obviously, a dedicated material should be used for model checking, but it can still provide important information about destruction of the part. As was demonstrated by uniaxial stretching, the first signs of permanent damage of the material appear inside of the material, and not on its surface.

## 5. Conclusions

The main goal of our investigation was to prove that stress whitening observations may be applied to evaluate the residual stresses in injection molded products. This aim was accomplished: a diversity of the stress profiles across the samples could be observed. The method was partially confirmed by photoelasticity and residual stress formation in injection molding. The results are promising and could be used in future research. Despite its potential, it still needs to be refined and developed. The biggest issue at the moment is obtaining a proper and reliable gray scale to elongation (*G-ε*) calibration curve.

We hope that this method, after several adjustments, will find its place among other residual stress measurement techniques and may provide insights into the effects of stress formations in parts, mainly in the cases of injection molding and extrusion.

## Figures and Tables

**Table 1:** Injection molding parameters.

Parameter	Value
Cylinder temperature	220–250 °C
Mold temperature	60 °C
Injection speed	35 cm^3^/s
Cooling time	25 s
Holding pressure	20 MPa, 60 MPa, 100 MPa
Holding time	4 s

**Table 2:** Calibration of the grey scale to elongation (*G-ε*) curve parameters used in calculation.

Parameter	Value
*A*	69.2
*B*	147
*C*	179
*D*	1.82
